# Towards a ‘clicked’ PSMA targeting gene delivery bioconjugate-polyplex for prostate cancer†

**DOI:** 10.1039/d4ra03640a

**Published:** 2024-07-29

**Authors:** Amanda R. Noble, Saeed Akkad, Nicholas D. J. Yates, James M. Jeffries, Nathalie Signoret, Martin A. Fascione

**Affiliations:** a Department of Chemistry, University of York Heslington York YO10 5DD UK martin.fascione@york.ac.uk; b Hull York Medical School, University of York York YO10 5DD UK nathalie.signoret@york.ac.uk

## Abstract

Prostate cancer is the most common cancer in men in the UK with over 50 000 new cases diagnosed each year and although therapeutic advances in surgery, anti-androgens, radio- and chemotherapy have increased survival rates, there still remains a need for new treatments to combat the most aggressive forms of the disease. Gene therapy offers promise as an alternative approach but is reliant on selective targeting to the cancer cell surface. Herein we describe the novel construction of a prostate specific membrane antigen (PSMA) binding bioconjugate-polyplex, based on a glutamate–urea peptide scaffold using ‘click’ chemistry, which we demonstrate is capable of targeted delivery of a GFP gene to PSMA overexpressing prostate cancer cells, and therefore may have potential future application as part of a prostate cancer gene delivery therapy.

## Abstract 2

We describe the construction of a prostate specific membrane antigen (PSMA) binding bioconjugate-polyplex, using ‘click’ chemistry, which we demonstrate is capable of targeted delivery of a GFP gene to PSMA overexpressing prostate cancer cells.

## Graphical abstract

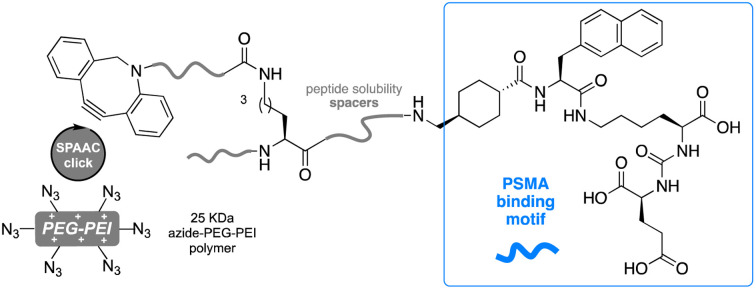

## Introduction

1 in 8 men suffer from prostate cancer (PCa) in their lifetime, and as such it is the most common cancer in men in the UK, with over 50 000 new cases diagnosed each year.^1^ PCa is a highly complex heterogenous cancer, which emanates from the prostate gland and has many classifications including aggressive, nonaggressive, high-grade and low-grade that allow treatment to be selected accordingly.^2^ In contrast to localised PCa which is frequently treated by radical prostatectomy, advanced forms of PCa require alternative methods capable of addressing the intrinsic changes of the cells.^3^ These typically include taxane-based chemotherapies (docetaxel) and second-generation anti-androgens (enzalutamide).^4,5^ Although these approaches can slow progression of PCa, the 5 year survival rate is still only ∼50% when the cancer is diagnosed at a late stage,^6^ meaning there is still an urgent need for new therapeutic approaches.

Gene delivery^7,8^ for treating cancer holds great promise with examplars^9–14^ like Gendicine (RAd-p53), which delivers the p53 tumour suppressor gene by an adenoviral vector, used for the treatment of several cancers. However, there are many more examples of failed treatment including Prostvac,^15^ a PCa immunotherapy delivered by poxviral vectors containing transgenes for the prostate specific antigen, which despite reaching stage 3 clinical trials was found to have no effect on overall survival. Traditional viral delivery systems suffer from well-established limitations including immunogenicity of the viral vector, payload constraints and potential interruption of essential genes through genomic integration.^16^ Alternatively, non-viral approaches^17^ including the use of cationic polymers, calcium phosphate and cationic lipids or combinations of these, can offer advantages such as increased payload capacity and enhanced immunocompatibility. However, potential issues of chemical toxicity, variable transfection efficiency and off-target binding still exist. As such target directed methods of delivery are highly desirable and can localise polyplexes to cancer cell surface antigens, reducing toxicity and increasing transfection efficiency in the process. Prostate specific membrane antigen (PSMA) is one such cell surface antigen that it is overexpressed on prostate cancer cells and correlates with increasing disease severity.^18^ PSMA, a transmembrane glycoprotein with no known endogenous ligand, was first identified in 1987 by Murphy and co-worker^19^ who raised the monoclonal antibody 7E11-C5 after immunising mice with LNCaP cells (a prostate cell line). This led to the development of ProstaScint® for PCa imaging using radiolabelled 7E11-C5,^20^ and although this was a significant breakthrough in the field it was later discovered that this antibody only detected binding to intracellular PSMA of necrotic cells. Antibodies to the extracellular portion^21^ of PSMA were subsequently developed and the mAb J591 was radiolabelled and tested in clinical trials^22,23^ but found to have poor clearance. Since then a focus on smaller targeting agents has led to the discovery of a number of nanobodies,^24,25^ peptides^26^ and small molecules^27^ that can target PSMA with varying levels of success,^28,29^ including polyplexes for delivery of the apoptosis inducing TRAIL gene.^30^ In particular theranostics containing glutamate–urea motifs have exhibited high specificity and affinity for PSMA in contrast to other functional groups. This is exemplified by peptide PSMA617,^31^ a scaffold used in the clinically approved Lu-177 radio pharmaceutical treatment Pluvicto®,^32^ and the Ga68 PET imaging agent Locametz.^33^ Although much work has clearly been carried out in this area there are still few new treatments reaching the clinic. Herein we describe the novel construction of a PSMA binding polyplex based on a glutamate–urea peptide scaffold using ‘click’ chemistry, which we demonstrate is capable of targeted delivery of a GFP gene to PSMA overexpressing PCa cells, and therefore may have potential future application as part of a PCa gene delivery therapy.

## Results and discussion

**Fig. 1:**
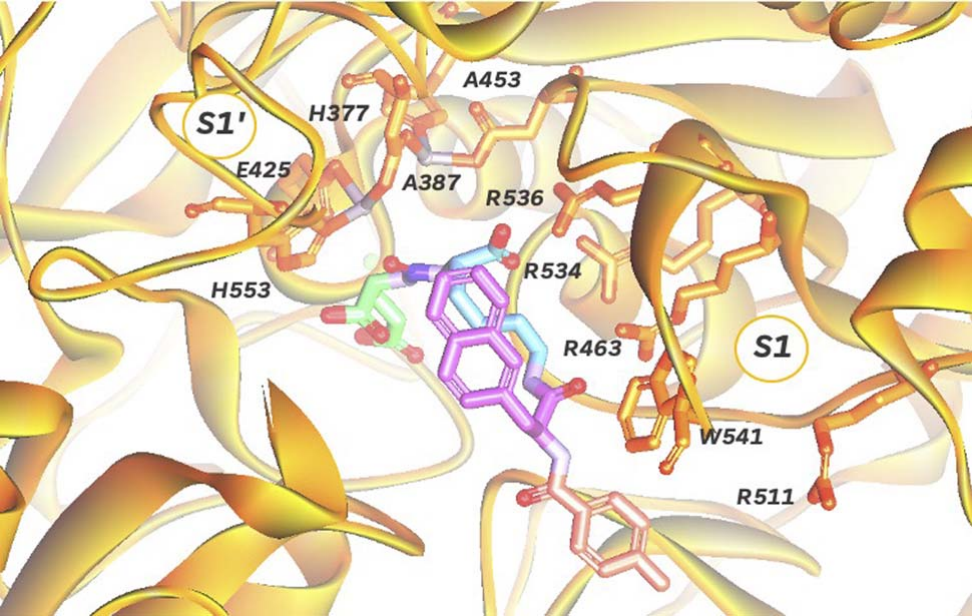
Depiction of PSMA dinuclear zinc active site cavity (orange, PDB: 5O5T) with a bound PSMA617 ligand, consisting of a glutamate (green)-urea (purple)-lysine (blue)-napthylalanine (pink)-tranexamic acid (pale red) motif.

**Fig. 2:**
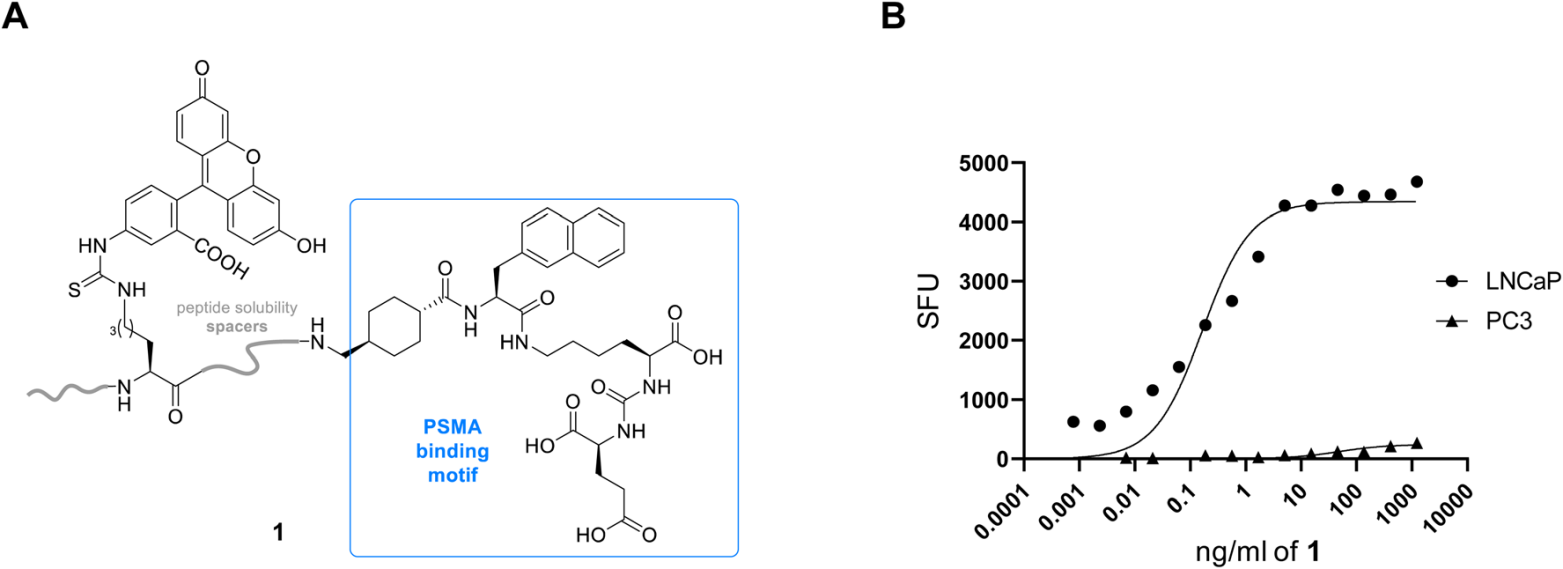
(A) Fluorescent anti-PSMA peptide 1 with characteristic Glu–urea binding motif. (B) Scatter plot demonstrating binding of fluorescent 1 to LNCaP (PSMA high) and negligible binding to PC3 (PSMA low) prostate cancer cell lines, following incubation for 90 min at 4 °C. SFU = specific fluorescence units.

In order to target PSMA specifically using a polyplex we opted to employ the high affinity signature motif of PSMA617. This motif consists of a glutamate–urea attached through a lysine to an unnatural hydrophobic napthylalanine and a tranexamic acid which binds the binuclear zinc active site of PSMA (Fig. 1). The active site cavity can be subdivided into an S1′, S1 and an arene binding pocket. The former consists of residues His377, Asp387, Glu425, Asp453 and His553 that interact with the C-terminal glutamate and the urea carbonyl *via* both polar and non-polar interactions,^34^ whilst the S1 arginine patch (Arg 463, Arg534, and Arg536) also binds the carboxylate of the lysine residue.^35^ The arene binding pocket, formed by Trp541 and Arg511, can then be filled by hydrophobic groups affording significantly increased binding affinity.^36^ Therefore we set out to synthesise this motif using solid phase peptide synthesis (SPPS, Fig. S1†), using hydrophilic peptide spacers to increase solubility, and elaborated with a distal fluorescein by an isothiocyanate coupling to yield fluorescent PSMA binding peptide 1 (Fig. 2A). To confirm that this peptide was still able to target PSMA when derivatised as would be required in polyplex construction, we comparatively screened fluorescent 1 for binding to PCa cell lines with both high (LNCaP) and low (PC3) levels of surface PSMA^37^ (Fig. 2B). Serial dilutions of peptide 1 were incubated at 4 °C to prevent internalization, and binding to cells assessed using flow cytometry. As anticipated, we observed selective binding PSMA positive LNCaP cells (*K*_D_ = 0.085 nM), with negligible binding to PC3 cells. This high affinity binding compares favourably with other literature studies^27^ using glutamate–urea scaffolds to target PSMA,^38,39^ and surpasses the reported binding affinity of anti-PSMA nanobodies, such as JVZ007 (*K*_D_ = 27.4 nM).^24^

**Fig. 3:**
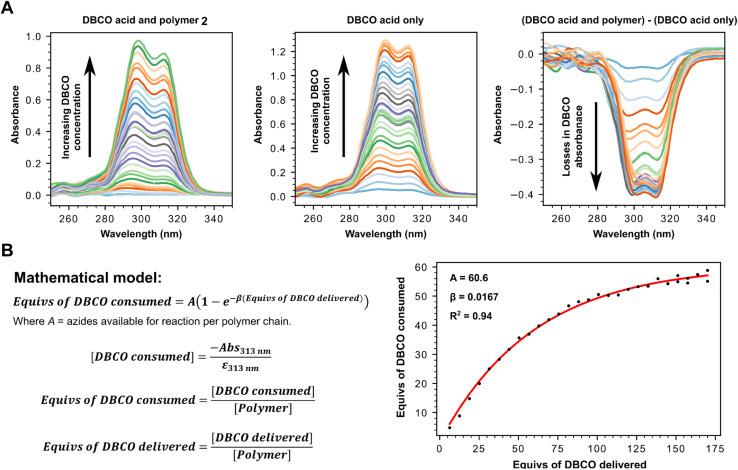
(A) UV-vis determination of number of reactive azides present on polymer 2 by monitoring the loss of absorbance from unreacted DBCO (313 nm) following click triazole formation. Stacked UV-vis spectra of click reaction between 0.571 μM azide containing polymer 2 (left) with increasing concentrations of DBCO-acid, stacked UV-vis spectra of DBCO acid only at equivalent increasing concentrations (centre), stacked UV-vis spectra for (Abs_313nm_ for DBCO acid and polymer) – (Abs_313nm_ for DBCO acid only) at different equivalent concentrations (right) to calculate ΔAbs_313nm_. (B) Mathematical model used to calculate number of reactive azides using ΔAbs_313nm_.

**Fig. 4:**
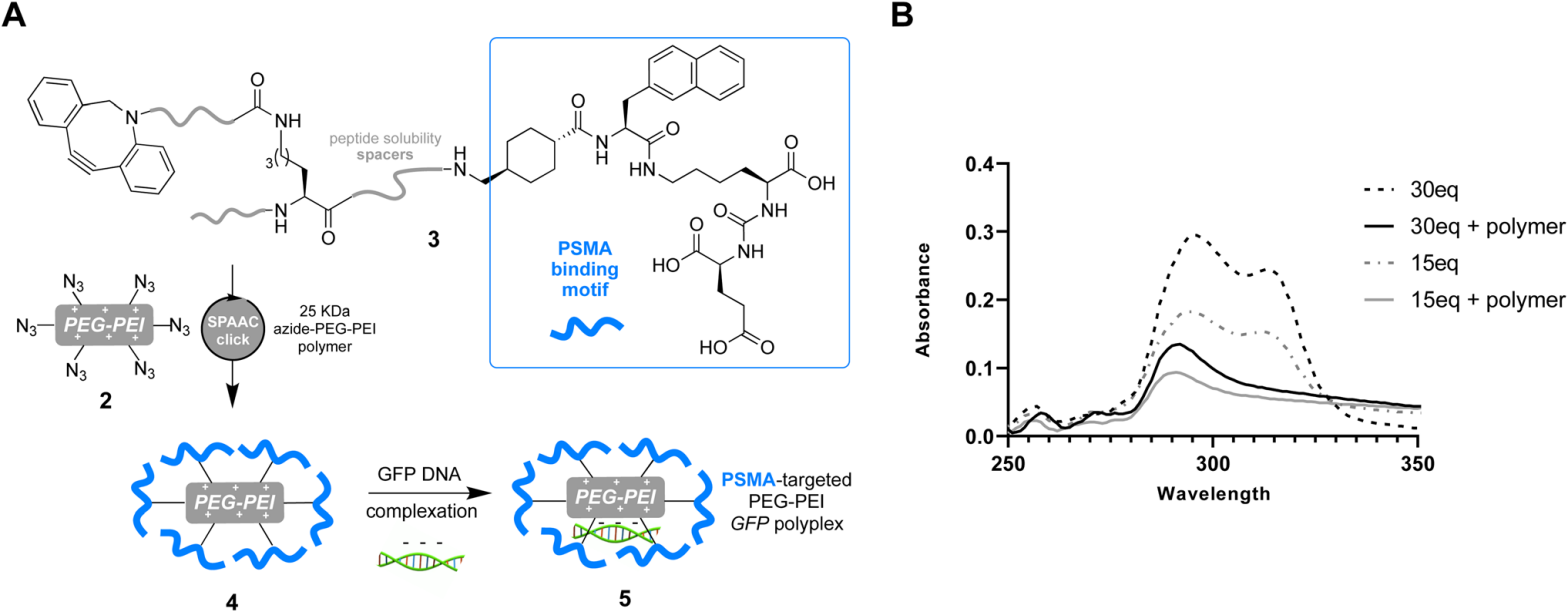
(A) Preparation of anti-PSMA-GFP polyplex *via* SPAAC ‘click’ reaction between DBCO peptide 3 and PEG–PEI azide polymer 2 yields the PSMA targeting polymer 4 (with 15 or 30 PSMA binding motifs) before complexation with GFP encoding vector to afford polyplexes 5. (B) UV-vis spectra of reactions between 30 or 15 equivalents of 3 before incubation (hashed line) and after incubation with 1 equivalent of polymer 2 after 18 h in DMSO (solid line), demonstrating loss of characteristic DBCO signal at ∼310 nm as ‘click’ reaction between 2 and 3 proceeds.

Having successfully confirmed selective binding to PSMA presenting cells, we next set out to incorporate the PSMA binding motif into a polyplex for selective gene delivery by conjugation to positively charged polymers capable of complexing negatively charged DNA. We opted to use a polyethyleneimine (PEI) polymer^40^ for this purpose as these cationic polymers are widely used for transient transfection of mammalian cells due to their high efficiency and low immunogenicity, especially when modified with polyethyleneglycol (PEG), and are therefore available at low cost in a variety of sizes. Starting from a commercially available 25 kDa branched PEI co-polymer grafted with azide functionalized PEG 2, we first confirmed the number of accessible azides as by using strain-promoted alkyne–azide ‘click’ chemistry (SPAAC)^41^ with a dibenzocyclooctyne (DBCO) strained alkyne (Fig. 3, Tables S1 and S2†). Monitoring the characteristic loss of the DBCO UV/vis peak at 310 nm upon reaction with the azides in the polymer by sequential addition of DBCO indicated ∼60 azides were accessible for SPAAC reaction per polymer. We then constructed another PSMA targeting peptide 3 (Fig. 4A) using SPPS and adorned it with a PEG spaced DBCO alkyne, replacing the fluorescein, using an DBCO–NHS ester coupling (Fig. S5†), and then subjected the azide polymer 2 to ‘click’ modification for 18 hours in DMSO with either 15 or 30 equivalents of the DBCO-peptide 3. Once again, we used characteristic loss of the 310 nm DBCO UV/vis peak to monitor the progression of the reaction after incubation at room temperature (Fig. 4B) and isolated the newly modified PSMA targeting polymer 4 (with 15 or 30 PSMA binding motifs) by dialysis and lyophilisation.

**Fig. 5:**
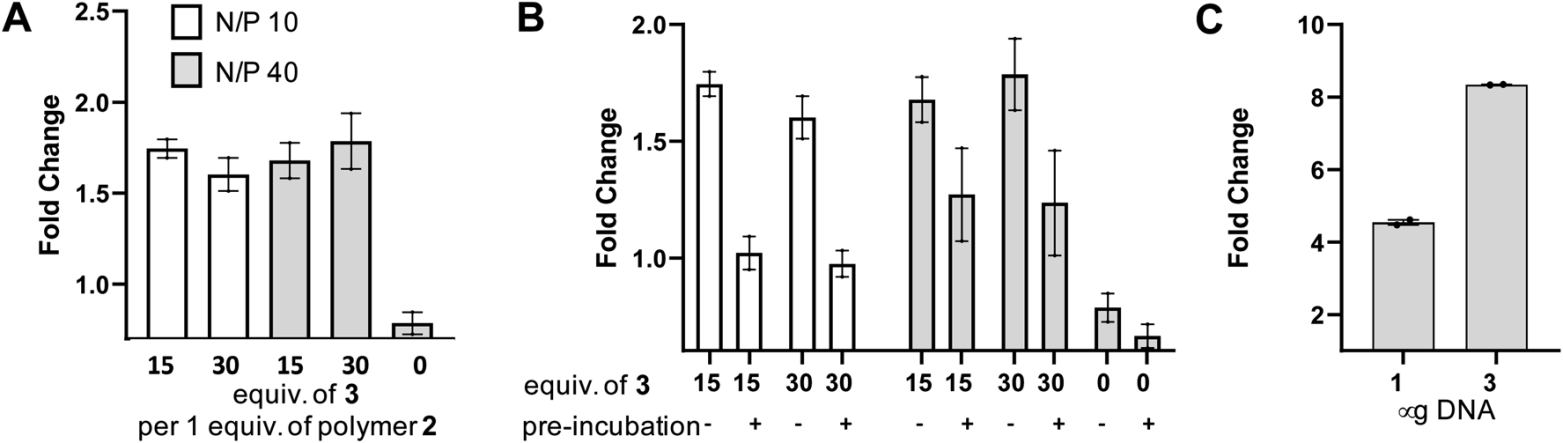
Delivery of GFP using anti-PSMA polyplexes 5. (A) Number of GFP positive cells by flow cytometry expressed as fold change over cells only, using either polyplexes constructed with 15 or 30 equiv. of 3 per 1 equiv. of azide polymer 2 (0 equiv. is polymer only), with GFP DNA associated in N/P ratios of 10 (white) and 40 (grey). DNA delivered at 1 μg ml^−1^ media. (B) PSMA antigen was blocked using S10 prior to addition of polyplexes. (C) Number of GFP positive cells by flow cytometry expressed as fold change over cells only when using polyplexes constructed with 30 equiv. of 3 per 1 equiv. of azide polymer 2 (N/P 40) at either 1 or 3 μg of DNA per ml media.

In order to test the utility of the PSMA targeting polymer 4 as a gene delivery agent we assembled polyplexes 5 (Fig. 4A) with a mammalian expression vector encoding a green fluorescent protein (GFP) gene under a CMV promoter, in amino to phosphate charge ratios (N/P)^42^ of 10 and 40. The PSMA targeting GFP polyplex was then delivered to LNCaP cells at an amount equating to 1 μg of DNA/ml of media. Media was changed 24 hours after transfection and the cells were harvested 96 hours later for flow cytometry analysis. Pleasingly, we observed an increase in the number of GFP positive cells (>1.5 fold change, Fig. 5A), for samples treated with N/P 10 or 40 polyplexes modified with PSMA binding peptide 3 (15 or 30 equiv.) compared to the cells only control, demonstrating that transfection of GFP had been achieved with PSMA targeting polyplexes 5. Importantly, negligible change in fluorescence was observed when using a polyplex lacking the PSMA binding peptide 3 (0 equiv.). To confirm that this observed increased GFP expression was a result of gene delivery with binding to cell surface PSMA, LNCaP cells were pre-incubated with a competing unmodified PSMA binding peptide (S10) to block cell surface PSMA binding sites. For each polyplex the delivery of GFP was indeed reduced by pre-incubation with the PSMA blocking peptide (Fig. 5B), indicative of PSMA receptor-mediated polyplex uptake and GFP gene delivery. Finally flow cytometry analysis also demonstrated a concentration-dependent increase in the number of GFP positive cells following treatment with the N/P 40 polyplex (Fig. 5C).

In conclusion, we have validated that ‘click’ chemistry construction of polyplexes targeting the overexpressed PSMA antigen on prostate cancer cell surfaces can be achieved *via* SPAAC modification of azide containing PEI–PEG co-polymer scaffolds. Notably, PSMA targeted PEI–PEG scaffolds have previously shown limited cytotoxicity in experiments using prostate cancer cells.^43,44^ The viability of these tools was confirmed using flow cytometry with specificity explored using PSMA blocking. Central to this approach was synthesis of a high affinity PSMA binding peptide modified with a reactive DBCO strained alkyne, the use of which can be easily translated to other nanosystems^17,45^ for targeted delivery to prostate cancer cells in the future.

## Data availability

The data supporting this article have been included as part of the ESI.†

## Author contributions

AN, SA, NDJY, JJ performed peptide synthesis and bioconjugations. AN and NDJY performed polyplex construction, and AN performed cellular assays and analysis. NS and MAF supervised the project. AN, NS and MAF wrote the manuscript and designed the study. All authors commented on the manuscript.

## Conflicts of interest

The authors declare no competing financial interest.

## Supplementary Material

RA-014-D4RA03640A-s001
