# Supplementary material for: Towards a ‘clicked’ PSMA targeting gene delivery bioconjugate-polyplex for prostate cancer
Source: RSC Adv. 2024 Jul 29;14(33):23796–801. doi: 10.1039/d4ra03640a (PMC11284900; doi:10.1039/d4ra03640a)
Supplement: RA-014-D4RA03640A-s001 [file RA-014-D4RA03640A-s001.pdf]

Electronic Supplementary Material (ESI) for RSC Advances.
This journal is © The Royal Society of Chemistry 2024

Towards a 'clicked' PSMA Targeting Gene Delivery Bioconjugate-Polyplex for Prostate Cancer

Amanda R. Noble[1], Saeed Akkad[1], Nicholas D. J. Yates[1], James M. Jeffries[1], Nathalie Signoret[2]* and Martin A. Fascione[1]*

[1]Department of Chemistry, University of York, Heslington, York, YO10 5DD, UK. [2]Hull York Medical School, University of York, YO10 5DD

Supporting Information

All chemicals, unless otherwise stated, were obtained from commercial sources. 1D NMR spectra were recorded on a Bruker Advance Neo 700 MHz spectrometer. 1H NMR chemical shifts reported (in ppm) are relative to HOD (δ 4.79) with D_2O as the solvent. High Performance Liquid Chromatography-Electrospray Ionization Mass Spectrometry (HPLC-MS) analysis of peptides was performed using a Dionex UltiMate® 3000 Ci Rapid Separation LC system equipped with an UltiMate® 3000 photodiode array detector probing at 250–400 nm, coupled to a HCT ultra ETD II (Bruker Daltonics) ion trap spectrometer, using Chromeleon® 6.80 SR12 software (ThermoScientific), esquireControl version 6.2, Build 62.24 software (Bruker Daltonics), and Bruker compass HyStar 3.2-SR2, HyStar version 3.2, Build 44 software (Bruker Daltonics) at the Centre of Excellence in Mass Spectrometry, York. All mass spectrometry was conducted in positive ion mode. Data analysis was performed using ESI Compass 1.3 DataAnalysis, Version 4.1 software (Bruker Daltonics).Samples were chromatographically analysed using a ThermoScientific C18, 2.6 µ (50 mm × 2.1 mm, Thermoscientific). Samples were prepared in 1 : 1 water : acetonitrile + 1% (v/v) formic acid. Water, 0.1% formic acid by volume (solvent A), and acetonitrile, 0.1 % formic acid (solvent B) were used as the mobile phase at a flow rate of 0.3 mL min^{-1} at 30°C. A multistep gradient of 13.0 min was programmed as follows: 5% B for 0 min, a linear gradient to 10% B over 1 min, a linear gradient to 30% B over 1 min, a linear gradient to 50% B over 2 min, a linear gradient to 90% over 1 min, 90% B for 5 min, linear gradient to 5% B over 1 min, 5% B for 2 min. Note that the multistep gradient finishes in 5% B in order to re-equilibrate the column.

Supplementary Figure 1, Synthesis of Fluorescent anti-PSMA peptide 1

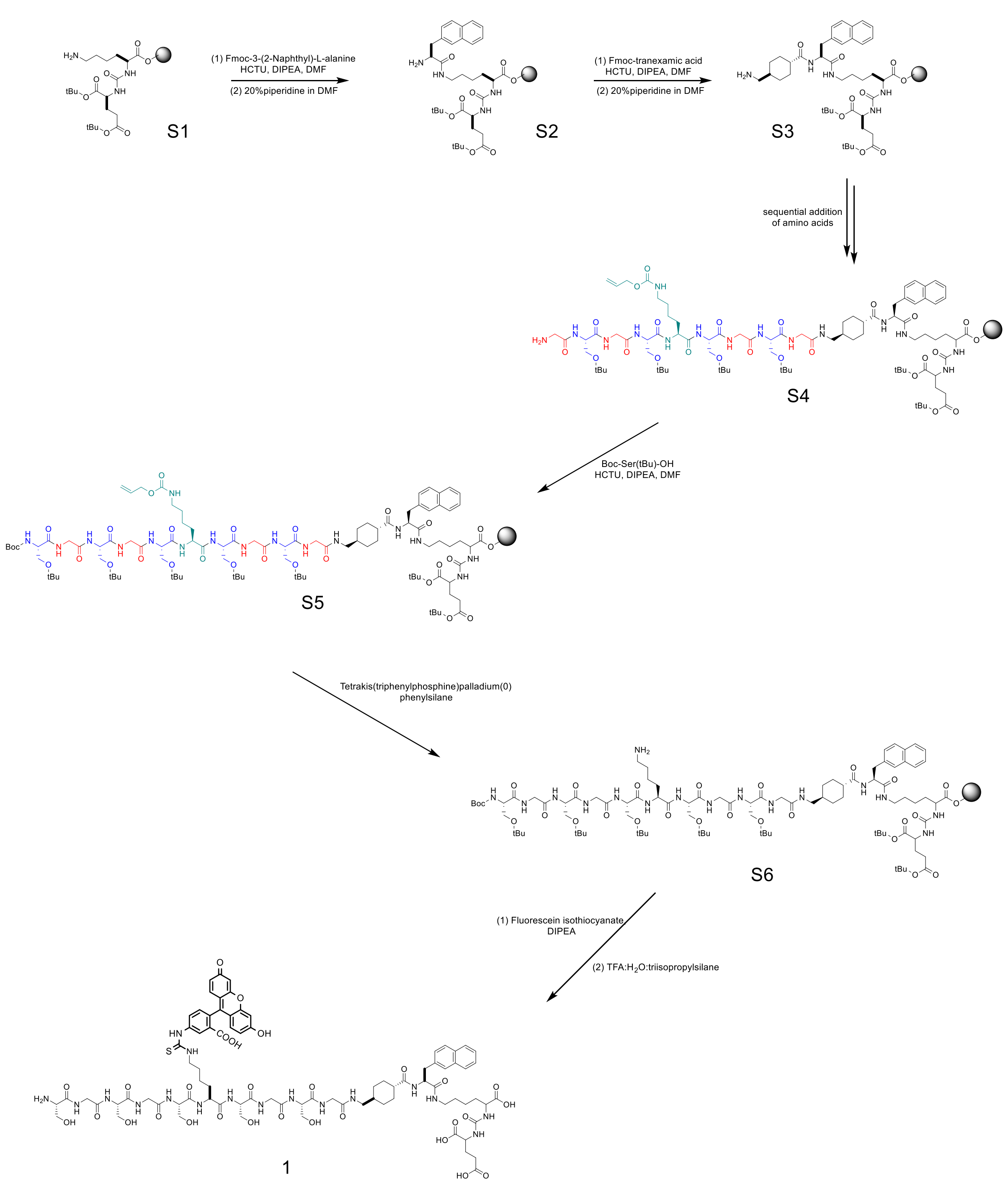

Synthesis of the linear anti-PSMA peptide S4

Solid phase peptide synthesis (SPPS) was used for making the resin-bound short peptide, adopted from Eder et al [1].

To 250 mg (0.305 mmol) of 2-chlorotrityl resin (swollen in dry DCM) was added 276 mg (0.61 mmol) of Fmoc-Lys(Alloc)-OH and 266 µL (1.53 mmol) of DIPEA (both dissolved in 3 mL of dry DCM). The mixture was left stirring at room temperature for 2 hours. The solution was then filtered out and the resin was washed two times with dry DCM followed by two washes (10 min each) with the capping solution DCM:MeOH:DIPEA (18:1.5:1.5 v/v). The resin was washed successively with DMF (5 x 2min), DCM, and MeOH (3 x 2min each) and dried under vacuum. The loading capacity of the resin was then quantified and found to be 0.67 mmol.g^{-1}.

The Fmoc group was then cleaved off by swelling the resin in DMF for 30 min and washing with 20% piperidine in DMF (5 x 2min). The resin was then washed successively with DMF (5 x 2min), DCM, and MeOH (3 x 2min each) and dried under vacuum.

For synthesising the glutamyl residue, the isocyanate species was generated in situ as follows: to an ice-cold solution of triphosgene (188 mg, 0.633 mmol) in 5 mL dry DCM was slowly added a mixture of H-Glu(OtBu)-OtBu (562 mg, 1.9 mmol) and DIPEA (1 ml) in 30 mL of dry DCM. The mixture was left stirring on ice for an hour followed by 2 hours at room temperature. The resin (315 mg, equating to 0.211 mmol of Fmoc-Lys(Alloc)-OH) was then added to the solution which was gently stirred overnight. The solution was then filtered out and the Alloc group was cleaved off by agitating the resin with tetrakis(triphenylphosphine)palladium(0) (61 mg, 0.053 mmol) and morpholine (469 µL, 5.3 mmol) in DCM. The resin was then washed with DCM (5 x 2 min) then MeOH (3 x 2 min) and dried under vacuum. The rest of the anti-PSMA moiety was synthesised using standard Fmoc chemistry: Fmoc-3-(2-Naphthyl)-L-alanine (461 mg, 1.05 mmol), HCTU (436 mg, 1.05 mmol), and DIPEA (404 µL, 2.32 mmol) were dissolved in 3 mL of DMF and the solution added to the resin. This mixture was gently agitated for 1 hour, then the resin was filtered and washed with DMF (3 x 2min). Fmoc deprotection was achieved by agitating the resin with a solution of 20% piperidine in DMF (5 x 2min). The resin was washed with DMF (5 x 2min) prior to the next coupling. The above steps were repeated for the addition of Fmoc-tranexamic acid (399 mg, 1.05 mmol).

A test cleavage was carried out on **S3** after addition of the tranexamic acid, with characterisation by HPLC-MS.

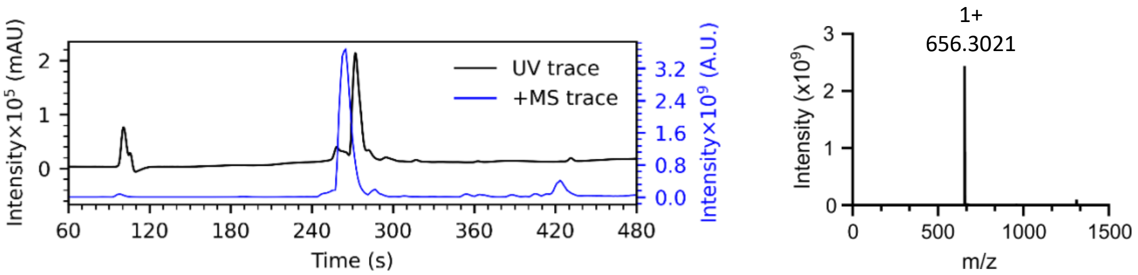

Figure S2. UV Abs 210-400 nm chromatogram with MS trace (left) and mass spectrum (right) of **S3** test cleavage analysed by HPLC-MS. ESI: Found $[M+H]^+$ 656.3021, $C_{33}H_{46}N_5O_9^+$ requires 656.3290.

The rest of the linear peptide **S4** was synthesised as above using the following order of the amino acids (1.05 mmol each): Fmoc-Glycine, Fmoc-Serine, Fmoc-Glycine, Fmoc-Serine, Fmoc-Lysine (Alloc), Fmoc-Serine, Fmoc-Glycine, Fmoc-Serine, Fmoc-Glycine.

The resin was finally washed with DCM, and MeOH (3 x 2min each) and was then dried under vacuum.

Synthesis of fluorescent anti-PSMA peptide 1

115 mg (0.077 mmol) of the linear anti-PSMA peptide resin **S4** was swollen in DMF for 30 min. Boc-Ser(tBu)-OH (101 mg, 0.385 mmol), HCTU (160 mg, 0.385 mmol), and DIPEA (148 µL, 0.847 mmol) were dissolved in 3 mL of DMF and added to the resin which was gently agitated for 1 hour. The solvent was then removed and the resin was washed with DMF (5 x 2min) and DCM (3 x 2min). The Alloc group was cleaved off by gently agitating the resin with tetrakis(triphenylphosphine)palladium(0) (22 mg, 0.019 mmol) and morpholine (170 µL, 1.92 mmol) in DCM for 1h. The resin was then washed with DCM and DMF (5 x 2min each) and filtered.

60 mg (0.154 mmol) of fluorescein isothiocyanate isomer I and 148 µL (0.847 mmol) of DIPEA was dissolved in 3 mL of DMF and added to the resin. The mixture was gently agitated in the dark overnight. The resin was then washed with DMF (5 x 2min) followed by DCM and MeOH (3 x 2min each) and was dried under vacuum overnight.

The target peptide was then cleaved off the resin by gently agitating the resin with a mixture of 95:2.5:2.5 (v/v) of TFA:H_2O:triisopropylsilane for 1 hour. The solution was added to ice-cold Et_2O to precipitate the peptide which was then centrifuged at 4000rpm 4°C for 10 min to give a pellet. The pellet was washed with cold Et_2O and centrifuged 3 times, then dissolved in 10% aq. acetic acid and freeze-dried overnight.

A final purification step was performed by dissolving the peptide in LC-MS grade water and loading it on a C18 solid-phase extraction (SPE) cartridge. The target peptide was eluted with 40% MeCN in water. The solution was lyophilised to give a bright yellow residue **1** (114 mg, 80%). HRMS-ESI: Found $[M+2Na]^{2+}$ 940.8344, $C_{83}H_{105}N_{17}Na_2O_{29}S^{2+}$ requires 940.8385.

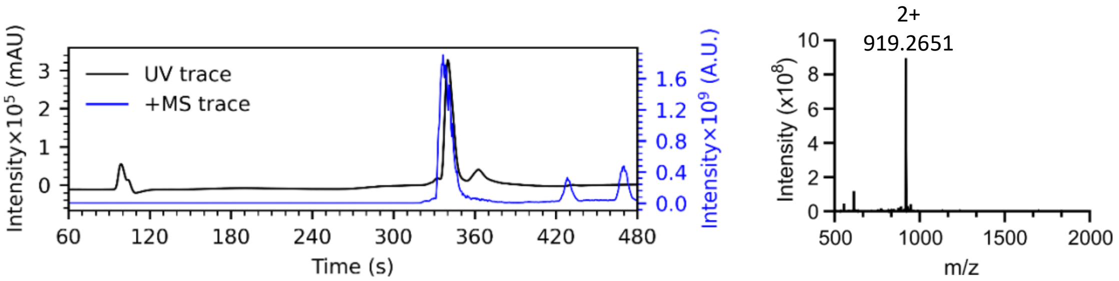

Figure S3. UV Abs 210-400 nm chromatogram with MS trace (left) and mass spectrum (right) of **1** measured by HPLC-MS. LRMS (ESI-pos): Found $[M+2H]^{+2}$ 919.2651, $C_{83}H_{106}N_{17}O_{29}S^{2+}$ requires 918.3529.

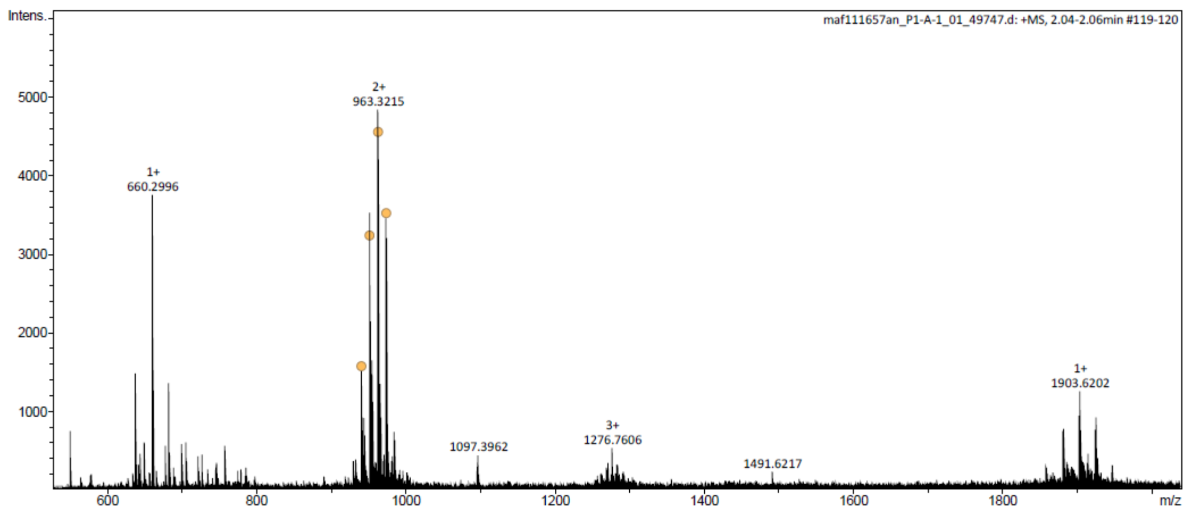

Meas. m/z	#	Ion Formula	m/z	err [ppm]	err [mDa]	mSigma	Mean err [ppm]
940.8344	1	C83H105N17Na2O29S	940.8385	4.3	4.1	49.5	2.8
951.8290	1	C83H104N17Na3O29S	951.8294	0.4	0.4	60.7	1.1
962.8191	1	C83H103N17Na4O29S	962.8204	1.4	1.3	37.9	1.0
973.8113	1	C83H102N17Na5O29S	973.8114	0.1	0.1	34.7	0.3

Figure S4 HRMS-ESI of **1**

Binding of Fluorescent anti-PSMA peptide 1 to prostate cancer cell lines (Figure 2B)

LNCaP and PC3 cells were detached using 10 mM EDTA in PBS, washed, re-suspended in ice cold PBS and plated in a 96 well plate at 75, 000 cells per well. Cells were incubated for 1.5 hour at 4 °C with **1** at serial dilutions (1:3) of 50 μg/ml –2.9×10^{-8} μg mL^{-1}. Cells were then washed, fixed overnight in FACS buffer (PBS 1% FCS, 0.05% sodium azide) containing 1% formaldehyde and resuspended in FACS buffer for analysis on a Cytoflex S (Beckmann Coulter). A Live/Dead stain was used on cells only for gating purposes. Median fluorescent units of cells only were subtracted from cells plus peptide giving specific fluorescent units. Data was analysed using GraphPad Prism, One site – Specific binding. The Kd was 0.1663 ng/ml, 0.085 nM.

Supplementary Figure 5, Synthesis of DBCO anti-PSMA peptide 3

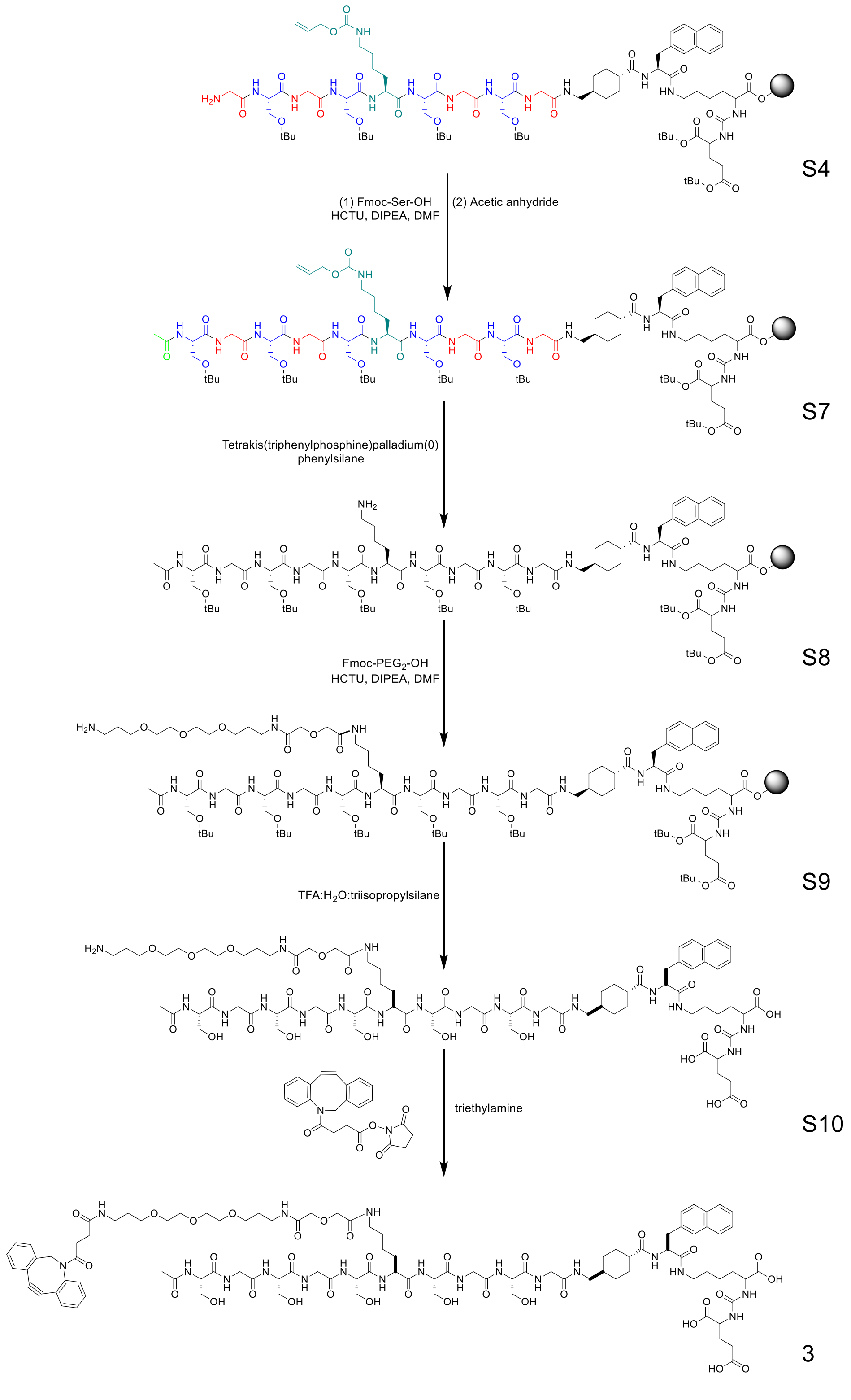

Synthesis of anti-PSMA peptide precursor S10

Synthesis of the linear peptide was repeated from **S1** (0.152 mmol) which was swollen in DMF. Then Fmoc-Ser(tBu)-OH (291.4 mg 0.76 mmol), HCTU (314 mg, 0.76 mmol), and DIPEA (293 μl, 1.67 mmol) were dissolved in 3 mL of DMF and added to the resin which was gently agitated for 1 hour. The solvent was removed and the resin was washed with DMF (5 x 2min). Fmoc deprotection was achieved by agitating the resin with a solution of 20% piperidine in DMF (5 x 2min). The resin was washed with DMF (5 x 2min) and capped with acetic anhydride (84.5 μl, 0.89 mmol), HCTU (314 mg, 0.76 mmol) and DIPEA (140 μl, 0.8 mmol).

The resin was then washed with DMF (5 x 2min) and DCM (3 x 2min). The Alloc group was cleaved off by gently agitating the resin with tetrakis(triphenylphosphine)palladium(0) (44 mg, 0.038 mmol) and phenylsilane (468 μl, 3.8 mmol) in DCM for 1h. The resin was then washed with DCM and DMF (5 x 2min each) and filtered. The resin was shrunk by washing with DCM (3x 2min) and MeOH (3x 2min) with rotation. The resin was dried initially on a vacuum manifold and then on high vacuum line overnight.

Roughly one third of this resin was taken and swollen in DMF for 30 min. Fmoc-PEG$_2$-OH (131 mg, 0.234 mmol), HCTU (97 mg, 0.233 mmol) and DIPEA (95 μl, 0.515 mmol) were dissolved in 1 ml DMF and added to the resin which was gently agitated for 2 hours. The resin was then washed with DMF (5 x 2min) followed by DCM and MeOH (3x 2min each) and was dried under vacuum overnight.

The target peptide was then cleaved off the resin by gently agitating the resin with a mixture of 95:2.5:2.5 (v/v) of TFA:H_2O:triisopropylsilane for 1 hour. The solution was added to ice-cold Et_2O to precipitate the peptide which was then centrifuged at 4000 rpm 4°C for 10 min to give a pellet. The pellet was washed with cold Et_2O and centrifuged 3 times, then dissolved in 10% aq. acetic acid and freeze-dried overnight affording a colourless solid **S10**. HRMS-ESI: found $[M+H+Na]^{2+}$ 915.4205, $C_{78}H_{123}N_{18}NaO_{31}^{2+}$ requires 915.4244.

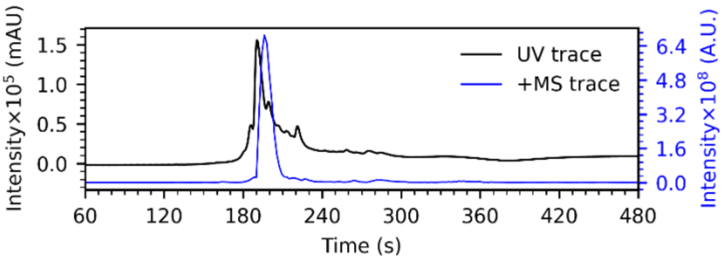

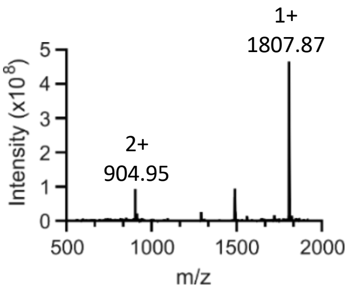

Figure S6. UV Abs 210-400 nm chromatogram with MS trace (left) and mass spectrum (right) of **S10** analysed by HPLC-MS. LRMS (ESI-pos): Found $[M+H]^+$ 1807.87, $C_{78}H_{123}N_{18}O_{31}^+$ requires 1807.86,

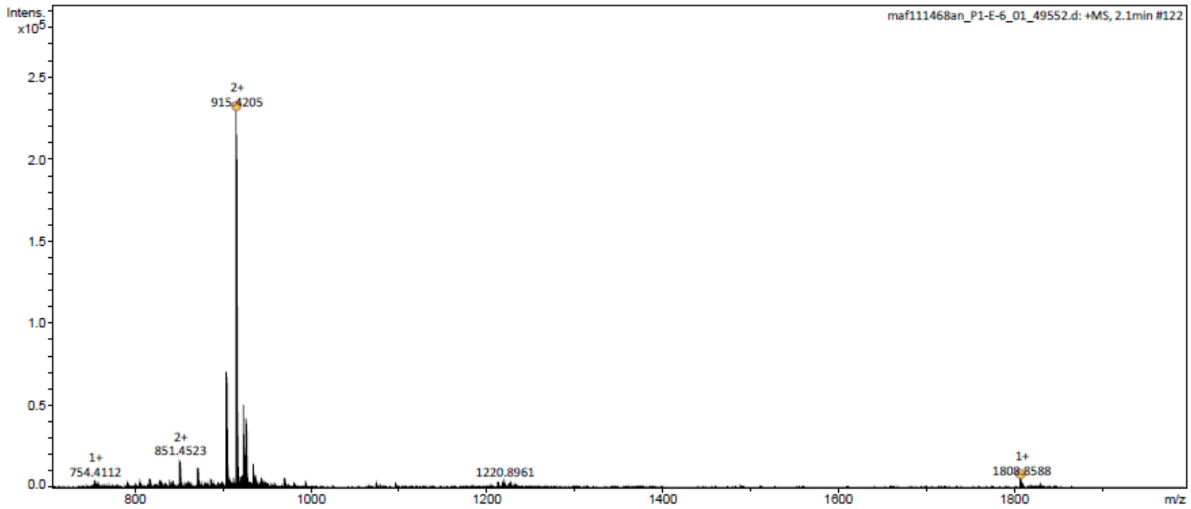

Meas. m/z	#	Ion Formula	m/z	err [ppm]	err [mDa]	mSigma	Mean err [ppm]
915.4205	1	C78H123N18NaO31	915.4244	4.2	3.9	10.0	3.9
1807.8547	1	C78H123N18O31	1807.8596	2.7	5.0	220.3	1.3

Figure S7 ESI HRMS of **S10**

Synthesis of anti-PSMA DBCO peptide 3

To 583 µl of a 3.3 mM solution of anti-PSMA PEG peptide **S10** in anhydrous DMF was added 3 equivalents of DBCO NHS ester (Sigma 761524) (159 µl of a 50 mM stock in DMF) and 1 µl of triethylamine. The resultant solution was stirred at room temperature overnight after which time it was diluted with water and lyophilised The lyophilised material was re-suspended in 5% DMF/water (2 ml), centrifuged and the supernatant was applied to a C18 column prewashed with methanol and then water. The column was eluted with a water/acetonitrile gradient. Fractions were checked by LCMS (**Figure S8**), the product **3** eluted in 40% acetonitrile/water and lyophilised to afford a colourless solid.

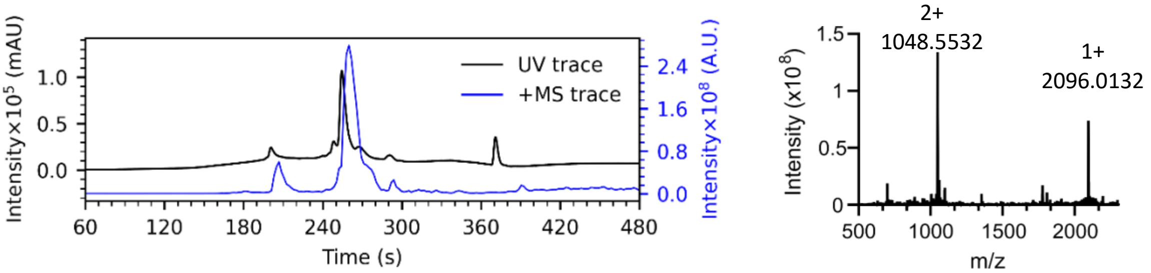

Figure S8. UV Abs 210-400 nm chromatogram with MS trace (left) and mass spectrum (right) of **3** measured by LC-MS. LRMS (ESI-pos): Found $[M+H]^{+}$ 2096.0132, $C_{97}H_{136}N_{19}O_{33}^{+}$ requires 2095.9576, Found $[M+H]^{2+}$ 1048.5532, $C_{97}H_{136}N_{19}O_{33}^{2+}$ requires 1048.4825

Calculation of the number of azides on PEI25K-g(20)-PEG3.4K-N3

A series of DMSO solutions were prepared such that the final volumes were 1 mL and the quantities of azide functionalised polymer **2** (PEI25K-g(20)-PEG3.4K-N3, Nanosoft Polymers) and DBCO-acid added to each solution were known. This enabled the concentrations of each of these species in the solution to be calculated. One series of solutions contained both DBCO-acid and polymer (see Table S1 below), whereas the other series lacked polymer but contained akin concentrations of DBCO-acid (see Table S2 below). The solutions were incubated overnight at rt in darkness, after which time the UV-vis spectra of the solutions were recorded.

DBCO-acid

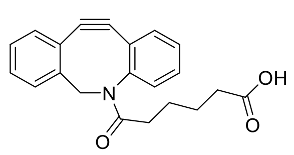

Unreacted DBCO absorbs strongly at 313 nm, whereas the triazole product of the reaction of DBCO with an azide does not. By comparing the absorbance at 313 nm between comparable solutions in the two series, it is therefore possible to obtain a value for $\Delta \boldsymbol{Abs_{313\,nm}}$. This value could then be used to determine much DBCO-acid has been consumed in each “Polymer and DBCO-acid” solution using the Beer–Lambert law $Abs = \varepsilon cl$, where $\boldsymbol{\varepsilon_{DBCO\ acid\ at\ 313nm}}$ was determined experimentally to be 14000 $dm^3\ mol^{-1}\ cm^{-1}$, and l was 1 cm.

$$\Delta \boldsymbol{Abs_{313\,nm}} = (\boldsymbol{Abs_{313\,nm}\ Polymer\ and\ DBCO acid}) - (\boldsymbol{Abs_{313\,nm}\ DBCO acid\ only})$$

$$[\boldsymbol{DBCO\ consumed}] = -\frac{\Delta \boldsymbol{Abs_{313\,nm}}}{\boldsymbol{\varepsilon_{DBCO\ acid\ at\ 313nm}}}$$

We define the equivalents of DBCO delivered to the “Polymer and DBCO-acid” solutions as

$$\boldsymbol{Equivalents\ of\ DBCO\ delivered} = \frac{[\boldsymbol{DBCO\ delivered}]}{[\boldsymbol{Polymer}]}$$

and we define the equivalents of DBCO consumed per polymer chain as

$$\boldsymbol{Equivalents\ of\ DBCO\ consumed} = \frac{[\boldsymbol{DBCO\ consumed}]}{[\boldsymbol{Polymer}]}$$

Ideally, the maximum equivalents of DBCO that could be consumed per polymer chain would be expected to equate to the quantity of azides present on each polymer chain. After DBCO is delivered to the polymer solutions and the resultant solutions are incubated for a fixed time period, the equivalents of DBCO consumed will therefore tend towards the number of azides present on each polymer chain. This behaviour will take the form of an exponential function of the equivalents of DBCO that were delivered to the “Polymer and DBCO-acid” solutions:

$$\boldsymbol{Equivs\ of\ DBCO\ consumed = A(1 - e^{-\beta(Equivs\ of\ DBCO\ delivered)})}$$

Where A = the number of azides available for reaction per polymer chain, and β is an undefined pre-exponential factor.

Non-linear regression of a plot of $\boldsymbol{Equivs\ of\ DBCO\ consumed}$ vs $\boldsymbol{Equivs\ of\ DBCO\ delivered}$ can therefore be used to determine the value of A, and by extension, the number of azides available for reaction per polymer chain.

Table S1: The concentrations of species present in samples of Polymer and DBCO-acid in DMSO, and the absorbance of these solutions at 313 nm.

Polymer and DBCO-acid sample	Concentration of Polymer / µM	DBCO-acid concentration delivered / µM	$Abs_{313\ nm}$
1 (Only Polymer in DMSO)	0.571	0	0.005
2	0.571	3.599	0.012
3	0.571	7.199	0.030
4	0.571	10.798	0.033
5	0.571	14.398	0.042
6	0.571	17.997	0.052
7	0.571	21.597	0.076
8	0.571	25.196	0.099
9	0.571	28.796	0.118
10	0.571	32.395	0.158
11	0.571	35.995	0.186
12	0.571	39.594	0.219
13	0.571	43.194	0.254
14	0.571	46.793	0.282
15	0.571	50.393	0.321
16	0.571	53.992	0.366
17	0.571	57.592	0.402
18	0.571	61.191	0.455
19	0.571	64.791	0.504
20	0.571	68.390	0.539
21	0.571	71.990	0.582
22	0.571	75.589	0.631
23	0.571	79.189	0.661
24	0.571	82.788	0.725
25	0.571	86.388	0.753
26	0.571	89.987	0.811
27	0.571	93.587	0.851
28	0.571	97.186	0.890
25 (diluted 1 in 2)	0.286	43.194	0.385
26 (diluted 1 in 2)	0.286	44.994	0.412
28 (diluted 1 in 2)	0.286	48.593	0.460

Table S2: The concentrations of samples of DBCO-acid in DMSO and their absorbance values at 313 nm.

DBCO-acid sample	DBCO-acid concentration / µM	$Abs_{313\ nm}$
1 (Only DMSO)	0.000	0.000
2	3.599	0.054
3	7.199	0.108
4	10.798	0.165
5	14.398	0.212
6	17.997	0.264
7	21.597	0.311
8	25.196	0.366
9	28.796	0.420
10	32.395	0.458
11	35.995	0.526
12	39.594	0.565
13	43.194	0.618
14	46.793	0.655
15	50.393	0.706
16	53.992	0.754
17	57.592	0.797
18	61.191	0.840
19	64.791	0.884
20	68.390	0.932
21	71.990	0.973
22	75.589	1.027
23	79.189	1.061
24	82.788	1.103
25	86.388	1.159
26	89.987	1.189
27	93.587	1.223
28	97.186	1.244
25 (diluted 1 in 2)	43.194	0.629
26 (diluted 1 in 2)	44.994	0.652
28 (diluted 1 in 2)	48.593	0.716

Synthesis of anti-PSMA polymer 4

A 1 mM solution of **3** in DMSO was prepared alongside a 20 µM solution of PEI25K-g(20)-PEG3.4K-N_3 **2** (Nanosoft Polymers) in DMSO. 100 µL reactions were set up as detailed in **Table S3** below and incubated at room temperature overnight. The following day reactions were diluted with water to less than 10% DMSO and dialysed into water before freeze drying. Reactions were monitored by UV-vis (**Figure 4B**).

Table S3. Concentrations and volumes required for setting up 100 μl reactions

Sample	Volume of 1 mM DBCO solution delivered / μL	Volume of 20 μM polymer solution delivered / μL	Additional DMSO added / μL	Final DBCO concentration of DBCO / μM	Final conc of polymer / μM	Equiv Of DBCO to polymer
1	30	0	70	300		
2	30	50	20	300	10	30
3	15	0	85	150		
4	15	50	35	150	10	15

GFP Transfection of LNCaP cells using anti-PSMA polyplex 5

LNCaP cells were plated in a 6-well plate at 175,000 cells/well with 2 ml of R10 (RPMI + 10%FCS + 1% glutamine, 1% PenStrep) media /well 24 hour prior to transfection.

The freeze dried anti-PSMA polymer **4** was resuspended in OptiMem to give a solution of 3 μg/μl. Each polymer has approximately 581 nitrogens/positive charges, using this information GFP (pmax-GFP from Lonza) (1 μg/μl) was associated with the polymer to give N/P ratios of 40 and 10 (**Table S4)**.

The GFP and anti-PSMA polymer **4** were added to OptiMem separately, gently vortexed and incubated for 10 min then combined, mixed gently and incubated for a further 15min to allow the GFP:anti-PSMA polyplex **5** to form before use. 1 ml of media was removed from cells leaving 1 ml to which was added dropwise the GFP polyplex **5** (100 μl). This was incubated for 24 hour then media was changed and cells were harvested 96 hours post transfection using 10 mM EDTA in PBS. Cells were plated in a 96 well plate, spun, washed with FACS buffer (PBS 1% FCS, 0.05% sodium azide) and analysed by Flow Cytometry on a CytoFlexS on the same day as harvesting.

Table S4. Volume of stocks to use for preparation of **5**

μl of GFP stock 1 μg/ μl added to 50 μl of OptiMem	μl of anti-PSMA polymer **4** stock (3 μg/ μl) added to 50 μl of OptiMem	N/P ratio when both solns are combined
1	5	10
1	20	40

Blocking of anti-PSMA prior to transfection (Figure 5B)

Cells and GFP:anti-PSMA polyplexes were prepared as above however 60 min prior to transfection, anti-PSMA PEG peptide **S10** (6 μM in media) was added to cells to block the PSMA. After this time the experiment proceeded as above.

Increased Concentration - GFP Transfection of LNCaP cells using anti-PSMA polyplex 5, (Figure 5C)

GFP Transfection was repeated as above and also with 3 μg DNA and 60 μl of anti-PSMA polymer stock (3 μg/ml) in a total final volume of 100 μl of OptiMem.

Figure S9. 700MHz ^{1}H-NMR analysis of anti-PSMA polymer **4** synthesised using 30 equiv. of DBCO anti-PSMA peptide **3**

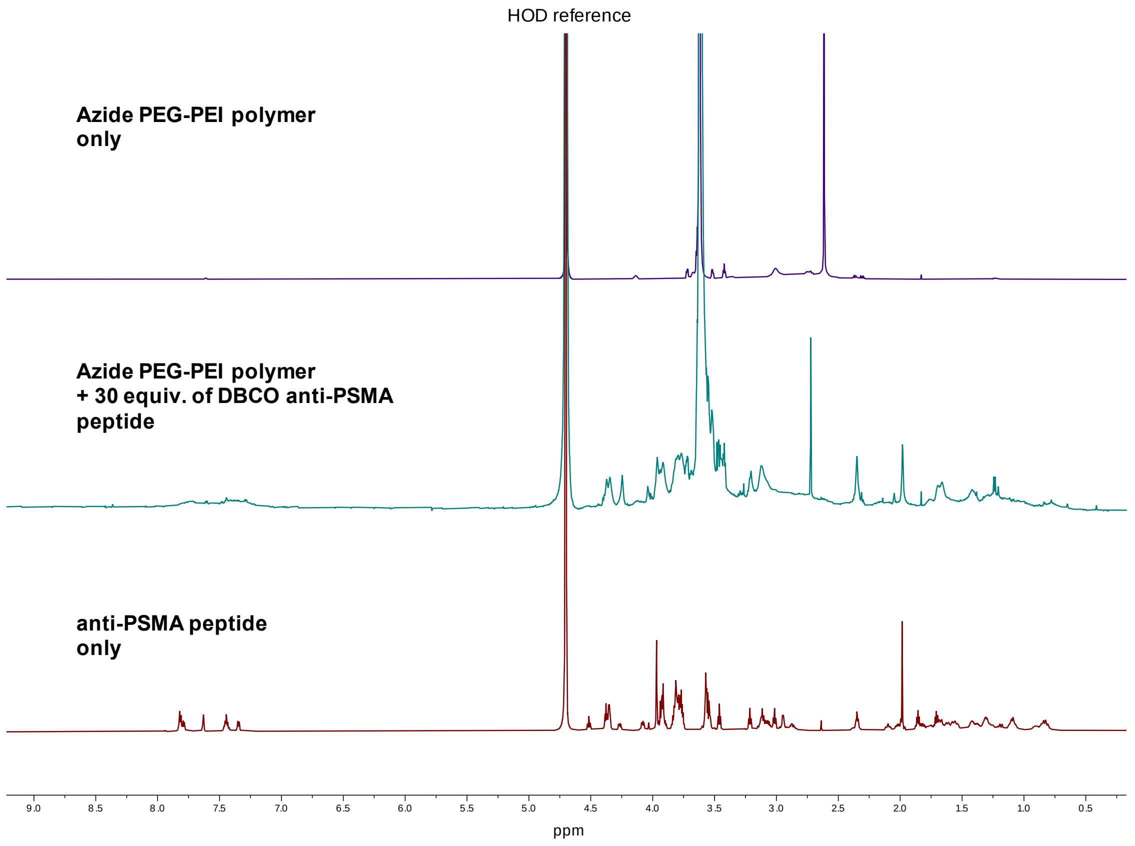

Figure S10. Expansion (3.2-4.5 ppm) of 700MHz ^{1}H-NMR analysis of anti-PSMA polymer **4** synthesised using 30 equiv. of DBCO anti-PSMA peptide **3**

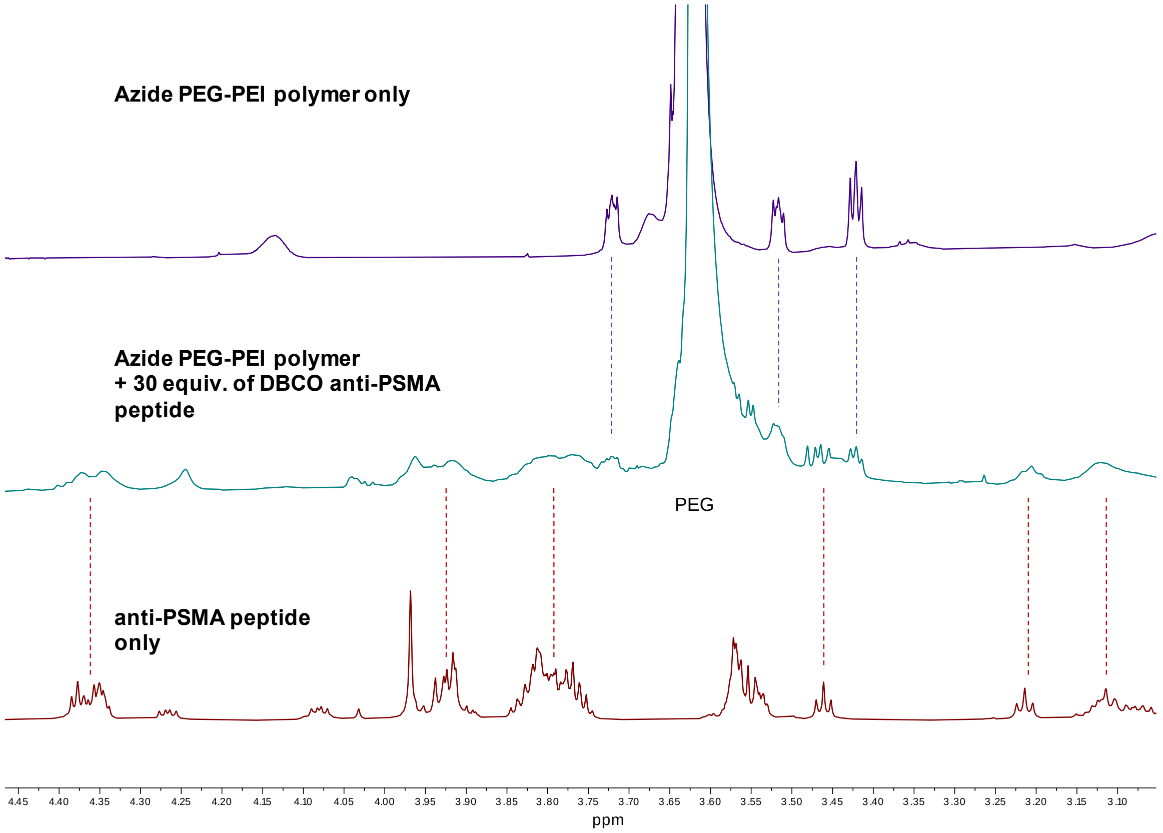

Figure S11. Expansion (3.3-8.0 ppm) of 700MHz ^{1}H-NMR analysis of anti-PSMA polymer **4** synthesised using 30 equiv. of DBCO anti-PSMA peptide **3**

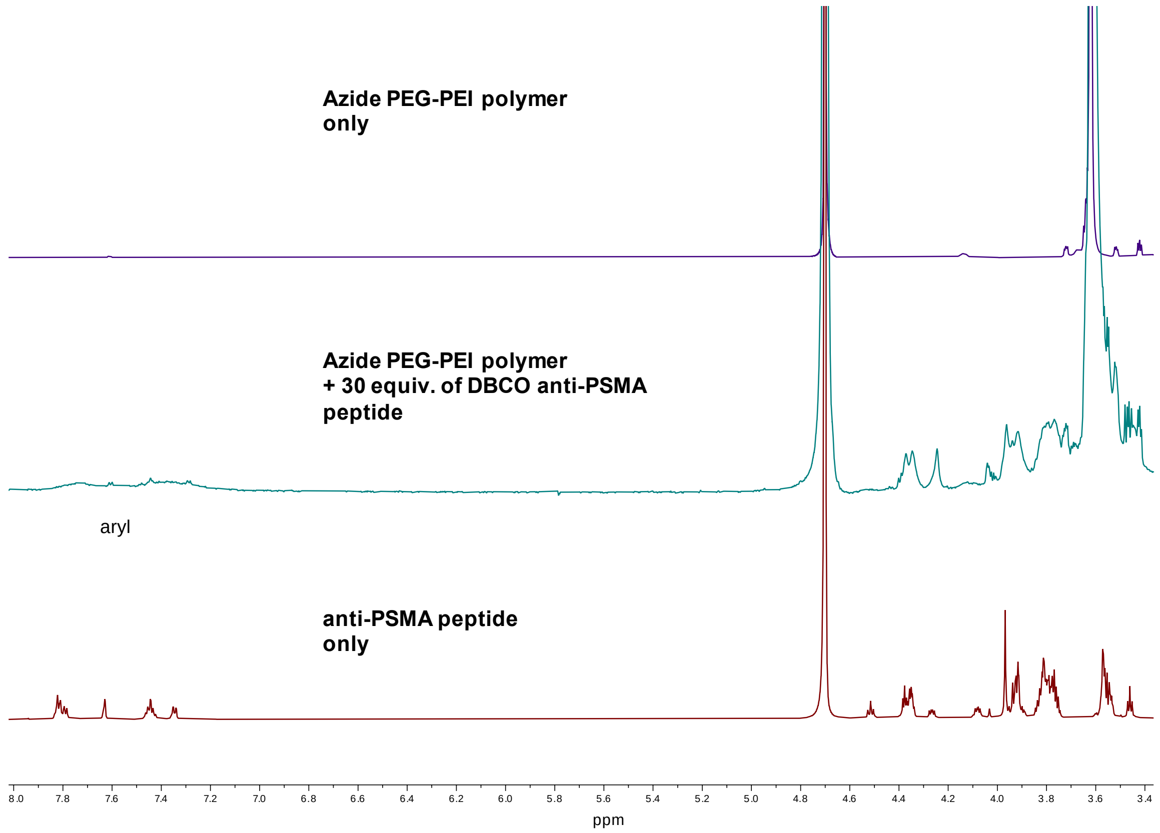

References

1 Eder, M. *et al.* 68Ga-Complex Lipophilicity and the Targeting Property of a Urea-Based PSMA Inhibitor for PET Imaging. *Bioconjugate Chemistry* **23**, 688-697 (2012).
